# In situ laser fenestration with coil embolization of expanding internal iliac artery aneurysm following previous stent graft coverage

**DOI:** 10.1016/j.jvscit.2026.102162

**Published:** 2026-01-27

**Authors:** Crista E. Horton, Jake Forman, Luc Lanteigne, Samantha Moore, Ahmed K. Ghamraoui, John J. Ricotta, Joseph J. Ricotta

**Affiliations:** a Department of Vascular Surgery, Delray Medical Center, Delray Beach, FL; b Department of Vascular Surgery, Florida Atlantic University Charles E. Schmidt College of Medicine, Boca Raton, FL

**Keywords:** Laser fenestration, Endoleak, Aneurysm, Iliac aneurysm, EVAR

## Abstract

We treated a large internal iliac artery (IIA) aneurysm from a type II endoleak with a prior endovascular aortic aneurysm repair and complete coverage of the IIA origin. In situ laser fenestration was used to create temporary access through the iliac limb, allowing selective coil embolization of feeding vessels and the aneurysm sac. The fenestration was then sealed with an iliac stent graft extension. This case demonstrates that in situ laser fenestration can provide a safe, fully endovascular option for managing complex type II endoleaks with IIA aneurysm expansion in high-risk patients.

In situ laser fenestration (ISLF) is an endovascular technique that uses laser energy to create controlled openings through stent graft fabric, allowing branch vessel revascularization in complex aortic arch repair.^1^ First reported by Murphy et al^2^ in 2009 for restoration of left subclavian artery (LSA) perfusion during thoracic endovascular aortic repair (EVAR), ISLF has since been applied for arch and visceral branch maintenance in select urgent and elective settings. Subsequent work shows that postfenestration dilation with noncompliant balloons minimizes graft tearing and creates an orifice suitable for stenting.^3^ Concurrent ISLF and LSA stenting during emergent thoracic EVAR has been reported as feasible, with low fenestration-related morbidity and excellent midterm patency.^4^ More recently, delayed ISLF LSA revascularization demonstrated 80% technical success without endoleak in patients with type B aortic dissection.^5^ Although ISLF is advantageous for branch preservation, long-term durability remains uncertain.^6^

Despite expanding experience, using ISLF to access an excluded internal iliac artery (IIA) to treat a type II endoleak and aneurysmal degeneration has not been described. Loss of IIA access poses a major technical challenge after EVAR with iliac limb extensions. We present a case in which ISLF was used to re-enter an excluded IIA to allow coil embolization of feeding branches and treatment of a large, rapidly expanding IIA aneurysm. This case and the images presented in this report were produced with the patient's consent.

## Case report

An 87-year-old man presented to the emergency department with mild, generalized abdominal pain. His history included hypertension, hyperlipidemia, atrial fibrillation on apixaban, prior EVAR for an infrarenal abdominal aortic aneurysm 20 years earlier, and subsequent left iliac limb extension into the external iliac artery with IIA coverage 1 year later. Both procedures were performed at an outside institution.

**Fig 1:**
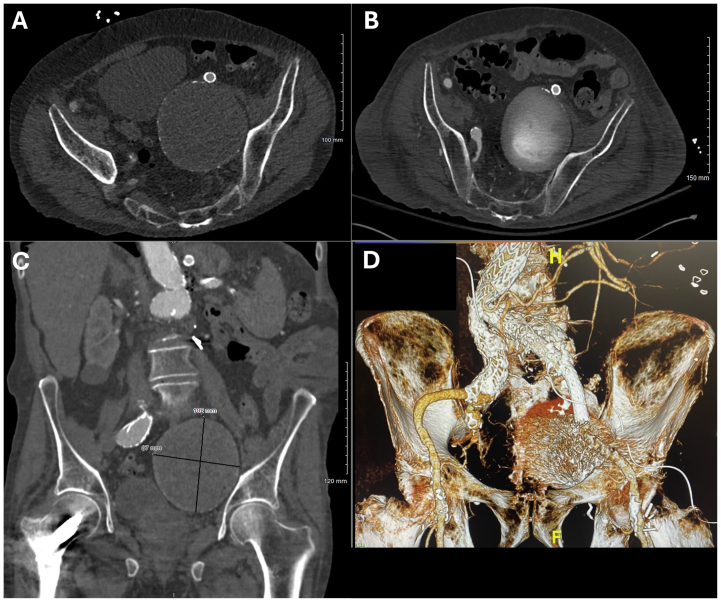
**(A-D)** Preoperative computed tomography (CT) imaging of a large left internal iliac artery (IIA) aneurysm after prior endovascular aortic aneurysm repair (EVAR). **(A)** Axial CT scan showing markedly enlarged left IIA aneurysm. **(B)** CT angiography (CTA) with contrast, consistent with a type II endoleak. **(C)** Coronal CTA demonstrating the aneurysm and retrograde filling. **(D)** Three-dimensional reconstruction.

Computed topography angiography (CTA) of the chest, abdomen, and pelvis demonstrated a large, left IIA aneurysm sac measuring 9.5 × 9.3 cm, representing >1 cm in growth from the prior year. Delayed phase imaging demonstrated persistent sac perfusion through collaterals from the right IIA and ipsilateral branches of the pudendal, epigastric, and circumflex iliac arteries, consistent with type II endoleak (Fig 1, *A*-*D*). The left IIA origin was covered by the prior limb extension. Device specifications were unavailable, but imaging suggested a polyester-based graft.

Given the patient's symptoms, frailty, rapid sac expansion, and sac size, intervention was recommended owing to rupture risk. Lack of access to the IIA origin required consideration of alternative approaches.

### Description of the procedure

**Fig 2:**
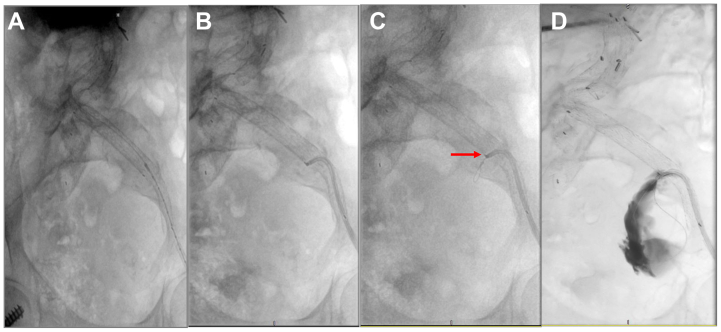
**(A-D)** In situ laser fenestration (ISLF) and access to the excluded left internal iliac artery (IIA) aneurysm. **(A)** Preprocedure diagnostic angiogram demonstrating large left IIA aneurysm. **(B)** A 6.5F steerable sheath positioned to achieve an optimal perpendicular angle for laser fenestration of the iliac limb. **(C)** Successful wire passage into the aneurysm sac to initiate fenestration. A *red arrow* indicates the location at which the laser breached the graft fabric. **(D)** Filling of sac with contrast after fenestration and before stent graft coverage and coil embolization.

Bilateral retrograde common femoral artery access was obtained. Two vascular closure devices were deployed in the left groin using a preclose technique, and the site was dilated for a 12F sheath. Diagnostic aortography from a 5F sheath in the right common femoral artery confirmed filling of the IIA sac via large collaterals from the ipsilateral branches of the pudendal, epigastric, and circumflex iliac arteries (Fig 2, *A*).

Intravascular ultrasound was advanced through the 12F left femoral sheath to identify the excluded IIA origin along the limb graft. A 6.5F steerable sheath was advanced and positioned at 90° against the graft wall (Fig 2, *B*). A 2.0-mm excimer laser catheter (Nexcimer, Philips) was used to perform ISLF of the iliac limb at a high fluence of 60 mJ/mm^2^ and frequency of 60 Hz, considering potential variation in graft fabric that could impact laser efficiency (Fig 2, *C* and *D*).

**Fig 3:**
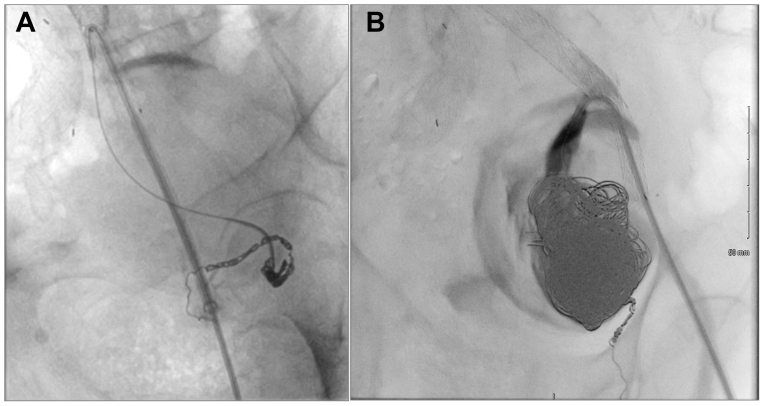
**(A** and **B)** Coil embolization of feeding vessels and aneurysm sac. **(A)** Embolization of collateral feeders using packing coils. **(B)** Completion coil embolization of the aneurysm sac until stagnant contrast was achieved.

After creating the fenestration, a 0.035-inch guidewire was advanced into the IIA, and the opening was dilated with a 4 mm × 40 mm balloon. Using a Lantern microcatheter (Penumbra), angled catheters, and 0.014-inch guidewires, we accessed the aneurysm sac and selectively embolized the inflow and outflow branches with appropriately sized coils (Fig 3, *A*). We then packed the lumen of the aneurysm sac with multiple large coils and confirmed thrombosis with angiography (Fig 3, *B*).

**Fig 4:**
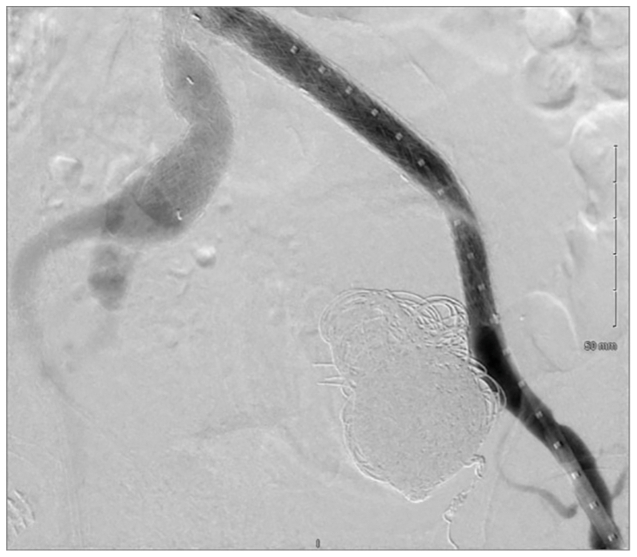
Postfenestration stent graft extension with completion angiography showing exclusion of the fenestration and absence of residual flow into the aneurysm sac.

After embolization, we sealed the fenestration with a 12 × 100 mm iliac limb extension deployed across the nascent opening with approximately 5 cm overlap on each side (W. L. Gore & Associates). The endograft was dilated post procedure with a compliant balloon for optimal seal. Completion angiography confirmed exclusion of the aneurysm, no residual endoleak, and preserved graft integrity (Fig 4).

**Fig 5:**
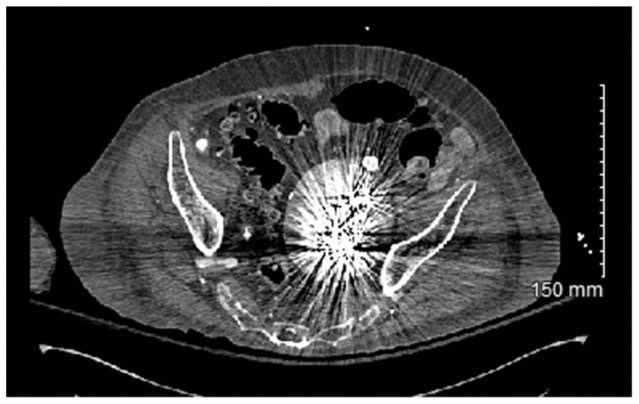
One-month postoperative computed tomography angiography (CTA) demonstrating a stable, thrombosed left internal iliac artery (IIA) aneurysm measuring 8.9 cm, with complete exclusion of type II endoleak.

The patient tolerated the procedure without complications. At the 1-month follow-up, CTA demonstrated a stable, bilobed IIA aneurysm measuring 8.9 cm, with no residual flow (Fig 5). Lifelong surveillance was planned with ultrasound examination every 6 months and ultrasound examination plus CTA for evidence of aneurysm growth or recurrence.

## Discussion

Intervention for type II endoleaks with sac expansion is indicated because the risk of rupture is increased in these circumstances. Endovascular management is first-line therapy; open conversion is reserved for unsuitable or failed endovascular approaches.^7^ Access becomes challenging when prior EVAR or limb extensions eliminate antegrade IIA entry, especially in high-risk patients.

Several alternative strategies exist, including translumbar, trans-sacral, and transarterial access, but anatomical constraints may preclude these approached. In this case, trans-sacral access was considered but rejected owing to difficulty reaching the sac and feeding branches, as well as potential sac pressurization. A chimney or snorkel approach from the contralateral side would require crossing the aortic bifurcation and introducing a wire and catheter between the graft and arterial wall, risking disruption of the distal seal zone, perforation, or embolization. Selective embolization of the feeding branches from the contralateral IIA was also considered, but rejected, owing to the added complexity over the selected approach. A transvenous approach was considered but rejected owing to risks of arterial-venous fistula formation, bleeding, and inadequate access to the large bilobed sac. Additionally, the use of shape memory polymer plugs is an alternative to reduce coil-related artifact on subsequent imaging; however, in this case our preference was to use standard coils.^8^ Ultimately, ISLF provided the most direct and controlled endovascular route, enabling secure access to the excluded IIA while preserving graft integrity.

ISLF has gained attention for branch vessel preservation in complex aortic repair, especially for LSA revascularization. Its off-label application to regain access to an excluded IIA, however, has not been reported previously. Although, Wheatley et al^9^ described bailout fenestration of the IIA using a re-entry device, to our knowledge, there are no other reports of ISLF used to facilitate coil embolization of an IIA aneurysm.

This case demonstrates that ISLF can be adapted safely to create temporary access to an excluded IIA, enabling embolization of a large aneurysm associated with type II endoleak. In patients unsuitable for open surgery, ISLF offers a fully endovascular option that maintains existing graft integrity while allowing definitive treatment.

## Conclusions

ISLF is an emerging technique adaptable beyond its traditional role in aortic arch and visceral revascularization. This case represents a novel application of ISLF to regain access to an excluded IIA and successfully treat a rapidly enlarging aneurysm owing to a type II endoleak after EVAR. In selected patients without feasible conventional access routes, ISLF provides a safe, fully endovascular alternative that avoids open repair while enabling definitive aneurysm exclusion.

## Funding

None.

## Disclosures

None.
